# Development of a method for adjusting trial results for biases in meta-analysis

**DOI:** 10.1186/1745-6215-14-S1-P124

**Published:** 2013-11-29

**Authors:** Rebecca Turner, Jelena Savovic, Hayley Jones, Jonathan Sterne, Nicky Welton, Julian Higgins

**Affiliations:** 1 MRC Biostatistics Unit, Cambridge, UK; 2 University of Bristol, Bristol, UK; 3 University of York, York, UK

## 

Randomised trials vary in methodological quality, and flaws in trial conduct can lead to biased estimation of the intervention effect. If a meta-analysis makes no allowance for methodological flaws, there's a danger that the results could be biased and over-precise, or that some lower-quality trials could be given too much influence in the meta-analysis. Cochrane review authors now use a specific ‘Risk of Bias' tool for assessing potential biases in the trials included in their review. This information is a valuable resource, but it is currently not clear how to use the ‘Risk of Bias' information to reduce the impact of suspected biases on the meta-analyses presented in the review.

In this work, we are developing a method for adjusting trial results for biases in meta-analysis, making use of ‘Risk of Bias' information for each trial and also generic external evidence on biases. We aim to integrate two existing methods for adjusting for bias in meta-analysis, in order to gain the advantages of both. One of the existing methods uses generic evidence on bias from an external collection of meta-analyses, while the other method relies on detailed assessment of the methodological quality of each trial and elicitation of opinion about the degree of bias likely to result. We consider several different approaches for integrating generic and trial-specific evidence on bias and discuss the practicalities of these.

